# 我如何诊断和治疗慢性粒-单核细胞白血病

**DOI:** 10.3760/cma.j.cn121090-20251217-00597

**Published:** 2026-02

**Authors:** 志坚 肖

**Affiliations:** 1 中国医学科学院血液病医院（中国医学科学院血液学研究所）、血液与健康全国重点实验室、国家血液系统疾病临床医学研究中心、细胞生态海河实验室，天津 300020 State Key Laboratory of Experimental Hematology, National Clinical Research Center for Blood Diseases, Haihe Laboratory of Cell Ecosystem, Institute of Hematology & Blood Diseases Hospital, Chinese Academy of Medical Sciences & Peking Union Medical College, Tianjin 300020, China; 2 天津医学健康研究院，天津 301600 Tianjin Institutes of Health Science, Tianjin 301600, China

## Abstract

慢性粒-单核细胞白血病（CMML）是一种同时具有骨髓增生异常和骨髓增殖特征的起源于造血干/祖细胞的髓系肿瘤。2022年第五版WHO诊断标准（WHO 2022）分类标准和国际共识标准（ICC）中对CMML的诊断、分型标准做了较大的修改。此外，在既往提出的CMML特异性预后积分系统（CPSS）基础上结合细胞遗传学和分子学新近又相继提出了CPSS-Mol、BLAST和BLAST-Mol等新的预后积分系统，CMML患者的治疗方案应在预后分组的基础上结合患者的症状、疾病负荷（血细胞减少或增殖表现）、疾病进展证据以及患者的个体状况和意愿综合考虑后做出。本文结合笔者诊治的病例对CMML的诊断和治疗要点与认识现况做了系统阐述。

## Abstract (en)

Chronic myelomonocytic leukemia（CMML）is one of myeloid neoplasms originating from hematopoietic stem/progenitor cells, featuring both myelodysplastic and myeloproliferative characteristics. Significant revisions have been made to the diagnostic and classification criteria for CMML in the 5th edition WHO classification（WHO 2022）and the International Consensus Classification（ICC）. Furthermore, besides the previously proposed CMML-specific prognostic scoring system（CPSS）, newer prognostic models such as CPSS-Mol, BLAST, and BLAST-Mol have recently been introduced, incorporating cytogenetic and molecular data. Treatment decisions for CMML patients should be comprehensively determined based on prognostic stratification, considering the patient's symptoms, disease burden（manifested as cytopenias or proliferative features）, evidence of disease progression, as well as individual patient status and preferences. This article provides an overview of diagnostic considerations, prognostic assessment and therapeutic options.

慢性粒-单核细胞白血病（Chronic myelomonocytic leukemia，CMML）是一种同时具有骨髓增生异常和骨髓增殖特征的起源于造血干/祖细胞的髓系肿瘤，其主要特征是外周血单核细胞持续增高［单核细胞绝对值（AMoC）≥0.5×10^9^/L且白细胞分类计数单核细胞比例≥10％］，骨髓一系或多系造血细胞发育异常，3～5年急性髓系白血病（AML）转化率15％～20％[1]–[5]。笔者结合诊治的病例对CMML的诊断和治疗要点与认识现况做一系统阐述。

例1，男，62岁，因“发现血小板减少2年”于2022年5月11日来我院就诊。查体肝、脾肋缘下未触及。外周血血细胞计数：WBC 4.64×10^9^/L，HGB 155 g/L，PLT 43×10^9^/L，AMoC 0.62×10^9^/L。外周血涂片分类计数：单核细胞13.4％，未见幼稚细胞。骨髓穿刺涂片：增生活跃，粒细胞占68.5％，红细胞占19％，粒∶红为3.61∶1，粒、红系细胞形态无明显异常，原始及幼稚单核细胞均未见，成熟单核细胞7％，全片共见巨核细胞343个。骨髓活检组织切片HE及PAS染色示骨髓增生较活跃（60％～70％），粒红比例大致正常，粒系各阶段细胞可见，幼稚细胞略增多，巨核细胞不少，分叶核为主。网状纤维染色（MF-1级，灶性）。染色体核型：46,XY[20]。血液系统疾病基因突变筛查全套：与疾病密切相关的热点突变位点有ASXL1 c.1927dupG p.G646Wfs*11 48.4％；CBL c.1139T>C p.L380P 1.7％；SRSF2 c.284C>A p.P95H 46％；TET2 c.4139A>T p.H1380L 92％。

修订的第四版世界卫生组织（WHO 2016）CMML诊断标准要求外周血AMoC≥1×10^9^/L，以及单核细胞比例≥10％[6]。在过去的近10年中关于CMML诊断的新认识主要有以下几个方面。

其一，2017年新提出了寡单核细胞CMML（oligomonocytic，OM-CMML）的概念，2019年CMML及其前驱疾病的维也纳专家共识提出了OM-CMML的最低诊断标准,除外周血AMoC为（0.5～0.9）×10^9^/L外，其他诊断条件同典型CMML最低诊断标准[7]。2022提出的国际共识标准（ICC）界定了意义未定的克隆性单核细胞增多症（CMUS）诊断标准[8]：①单核细胞持续增多，AMoC≥0.5×10^9^/L，单核细胞占白细胞比例≥10％；②不要求骨髓增生异常综合征（MDS）定义的血细胞减少［中性粒细胞绝对值（ANC）<1.8×10^9^/L，HGB<130 g/L（男）和<120 g/L（女），PLT<150×10^9^/L］；③存在至少一个髓系肿瘤相关基因突变且变异等位基因频率（VAF）≥2％；④血细胞无明显发育异常、无原始细胞（含幼稚单核细胞）增多或符合CMML的骨髓形态学表现；⑤不满足髓系或其他造血系统肿瘤的诊断标准；⑥无可解释单核细胞增多的反应性疾病。在符合CMUS的基础上，如果有血细胞减少则诊断为克隆性血细胞减少伴意义未定的单核细胞增多症（Clonal cytopenia with monocytosis of undetermined significance，CCMUS）。

其二，明确了用多参数流式细胞术（MFC）分析外周血单核细胞亚群分布异常在CMML诊断中的作用和地位。根据CD14和CD16的表达情况，单核细胞可进一步分为经典单核细胞（CD14强阳性/CD16阴性，MO1）、中间型单核细胞（CD14强阳性/CD16阳性，MO2）和非经典单核细胞（CD14弱阳性/CD16阳性，MO3）。当外周血中MO1单核细胞比例>94％对CMML诊断的敏感度超过90％，特异度超过95％[9]。这种外周血单核细胞亚型分布异常已被推荐为CMML的一个辅助诊断参数。

其三，CMML的分子异常谱系得以较完整解析，95％以上的CMML患者至少有1个及以上基因突变，包括DNA甲基化（TET2、DNMT3A、IDH1、IDH2）、RNA剪切（SRSF2、SF3B1、U2AF1、ZRSR2）、组氨酸修饰（ASXL1、EZH2）、细胞信号（NRAS、KRAS、CBL、NF1、PTPN11、JAK2）和转录因子（RUNX1、SETBP1、GATA2）等，最常见的突变基因是TET2、SRSF2和ASXL1，突变频率分别约为60％、50％和40％。大队列研究证实CMML常见的分子学特征改变是TET2-SRSF2共突变（～46％）和TET2双等位基因失活（biTET2，～45％）[10]–[11]。

基于上述认识进展，2022年第五版WHO诊断标准（WHO 2022）分类标准[12]（表1）和ICC[8]（表2）中对CMML的诊断、分型标准做了较大的修改。WHO 2022标准特别提出：①原始细胞及原始细胞等同细胞包括原始粒细胞、原始单核细胞和幼稚单核细胞。②骨髓增殖性肿瘤（MPN）在疾病过程中可能出现单核细胞增多，此种情况需与CMML鉴别。有明确MPN病史者应排除CMML，骨髓存在MPN特征和（或）高负荷MPN相关突变（JAK2、CALR或MPL）更倾向于支持诊断伴有单核细胞增多的MPN，而非CMML。③对于嗜酸性粒细胞增多的病例，需特别注意排除伴有酪氨酸激酶融合基因的髓系/淋系肿瘤。④形态学发育异常需在骨髓某造血系别中≥10％的细胞中出现。⑤外周血流式细胞术分析单核细胞亚群需在无已知活动性自身免疫性疾病和（或）系统性炎症综合征的情况下，检测到经典型单核细胞增多（>94％）。我们对按WHO 2016确诊的113例CMML和840例MDS按WHO 2022标准进行了重新诊断[10]：840例MDS患者中有19例符合WHO 2022 CMML诊断标准，既往诊断包括：3例MDS伴单系发育异常，5例MDS伴多系发育异常，1例MDS伴环状铁粒幼红细胞及单系发育异常，5例MDS伴原始细胞过多1型，5例MDS伴原始细胞过多2型；113例WHO 2016标准诊断的CMML患者有23例重新诊断为AML，包括18例AML伴NPM1突变（既往诊断包括3例CMML-0，4例CMML-1，11例CMML-2），3例AML伴KMT2A重排（既往诊断包括1例CMML-0，2例CMML-1）和2例AML伴MECOM重排（既往诊断包括1例CMML-0，1例CMML-1）。剩余90例患者符合WHO 2022 CMML诊断标准，既往诊断包括：40例CMML-0，28例CMML-1和22例CMML-2。

**表1:** WHO 2022慢性粒-单核细胞白血病（CMML）诊断分型标准

必要条件（所有均需满足）
①外周血单核细胞持续增多，单核细胞绝对值≥0.5×10^9^/L且单核细胞比例≥10％ ②外周血与骨髓原始细胞<20％ ③不满足CML或其他MPN的诊断标准 ④不满足伴有酪氨酸激酶融合基因的髓系淋系肿瘤的诊断标准
补充支持标准
– 如单核细胞绝对值≥1×10^9^/L，需满足至少1条支持标准 – 如单核细胞绝对值<1×10^9^/L且≥0.5×10^9^/L，需同时满足支持标准 ①和② ①至少1个髓系细胞系存在发育异常 ②获得性克隆性细胞遗传学或分子学异常 ③外周血单核细胞亚群分布异常
分型标准
– 骨髓增生异常型CMML：WBC<13×10⁹/L – 骨髓增殖型CMML：WBC≥13×10⁹/L
亚组标准
– CMML-1：外周血中原始细胞/幼稚单核细胞<5％，骨髓中<10％ – CMML-2：外周血中原始细胞/幼稚单核细胞5％～19％，骨髓中10％～19％

**注** CML：慢性髓细胞性白血病；MPN：骨髓增殖性肿瘤

**表2:** ICC 2022慢性粒-单核细胞白血病（CMML）诊断分型标准

诊断标准
①单核细胞绝对值≥0.5×10^9^/L，且单核细胞占白细胞比例≥10％ ②血细胞减少（标准同MDS） ③外周血、骨髓中原始细胞（包括幼稚单核细胞）<20％ ④存在克隆性证据^a^：异常染色体和（或）存在至少一个髓系肿瘤相关基因突变且变异等位基因频率（VAF）至少10％ ⑤骨髓细胞形态学检查结果符合CMML（因髓系增殖导致骨髓增生活跃，常伴单核细胞增加），且不符合AML、MPN或其他伴有单核细胞增多的疾病特征 ⑥无BCR::ABL融合基因，也无伴嗜酸性粒细胞增多和酪氨酸激酶融合基因的髓系/淋系肿瘤的遗传学异常
分型标准
– 骨髓增生异常型CMML：WBC<13×10⁹/L – 骨髓增殖型CMML：WBC≥13×10⁹/L
亚组标准
– CMML-1：外周血中原始细胞/幼稚单核细胞<5％，骨髓中<10％ – CMML-2：外周血中原始细胞/幼稚单核细胞5％～19％，骨髓中10％～19％

**注** ICC：国际共识标准；^a^无克隆性证据时必须满足：①单核细胞绝对值≥1×10^9^/L且占白细胞比例>10％；②原始细胞（含幼稚单核细胞）增多（骨髓中≥5％和/或外周血中≥2％），形态学发育异常，或符合CMML的异常免疫表型。MDS：骨髓增生异常综合征；AML：急性髓系白血病；MPN：骨髓增殖性肿瘤

由于WHO（2022）和ICC的CMML诊断标准均将外周血AMoC由原来的≥1.0×10^9^/L修订为≥0.5×10^9^/L，与反应性单核细胞增多的鉴别就显得尤为重要。常见的反应性单核细胞增多的原因有感染（细菌，病毒如HIV等，原虫如利什曼病等）、炎症性疾病（如类风湿性关节炎、系统性红斑狼疮等）、免疫性血细胞减少症和脾功能亢进等。MFC外周血单核细胞亚群、染色体核型（常规染色体核型和FISH）和二代测序基因突变分析寻找克隆性证据就成了鉴别诊断的关键。

例1外周血AMoC≥0.5×10^9^/L且单核细胞占白细胞比例≥10％，血小板计数减少，但骨髓和外周血细胞形态无明显细胞发育异常形态学改变，基因突变分析有ASXL1、CBL、SRSF2和TET2基因突变，最后确诊为CCMUS。

例2，男，25岁，因“发现脾大、血象异常1个月余”于2022年3月1日来我院就诊。查体：中度贫血貌，肝肋缘下未触及，脾肋缘下10 cm。血常规：WBC 16.88×10^9^/L，RBC 3.07×10^12^/L，HGB 72 g/L，MCV 78.5 fl，PLT 48×10^9^/L，白细胞分类计数单核细胞占比为16.5％，AMoC 2.79×10^9^/L。骨髓涂片：增生极度活跃，粒细胞占58.5％，红细胞占27％，原始单核细胞占0.5％，中晚幼粒细胞可见胞质颗粒减少，偶见双核粒细胞，中晚幼红细胞可见巨幼样变。全片共见巨核细胞73个。骨髓活检：HE及PAS染色示送检骨髓增生极度活跃（>90％），粒红比值增大，髓系幼稚阶段细胞略增多，巨核细胞不少，可见胞体小、分叶少的巨核细胞；淋巴细胞散在分布。网状纤维染色（MF-1级）。骨髓单个核细胞流式细胞术免疫表型分析：髓系原始细胞比例不高，CD117、CD38表达减弱，表型异常；粒系SSC减小，CD16表达缺失，部分表达CD38、HLA-DR，CD13/CD11b分化抗原表达异常；单核细胞比例偏高，占有核细胞的16.68％，CD14表达减弱，不表达CD300e，表型偏幼稚；B祖细胞罕见。外周血单核细胞亚群流式细胞术分析：经典单核细胞99.72％，中间型单核细胞0.28％，非经典单核细胞0％。染色体核型：46,XY[20]。基因突变检测与疾病密切相关的热点突变位点有：PHF6 c.751C>T p.Q251* 97.1％；U2AF1 c.101C>T p.S34F 46.8％；ASXL1 c.2143_2171dup p.R725Efs*10 8.1％；NRAS c.35G>A p.G12D 2.1％；KRAS c.35G>A p.G12D 3.5％；KRAS c.34G>A p.G12S 24.9％。诊断为：CMML（CMML-0期，MP-CMML，CPSS-Mol 4分高危）。

CMML的治疗决策，特别是是否启动造血干细胞移植，主要还是基于患者的预后危度分组。MDS的预后分组积分系统IPSS-R和IPSS-M都不适合CMML患者的预后分组。CMML较为共识的预后积分系统是CMML特异性预后积分系统（CMML-specific prognostic scoring system，CPSS）（表3）[13]，此后在CPSS基础上提出的CPSS-Mol[14]。CPSS-Mol根据是否有染色体核型异常及是否有ASXL1、NRAS、RUNX1和SETBP1突变的赋分（表4）的累计积分，将遗传学预后分为低危（0分）、中危1（1分）、中危2（2分）和高危（≥3分）等4个危度组。CPSS-Mol包括细胞遗传学预后分组（低危0分，中危1 1分，中危2 2分，高危≥3分）、骨髓原始细胞（<5％ 0分，≥5％ 1分）、白细胞计数（<13×10^9^/L 0分，≥13×10^9^/L 1分）和红细胞输注依赖（否0分，是1分）等4个预后参数，依累计积分，将患者分为低危（0分）、中危1（1分）、中危2（2～3分）和高危（≥4分）等4个预后危度分组，中位总生存（OS）时间分别为未达到、68、30和17个月，4年累积AML转化率分别为0％、8％、24％和52％。

**表3:** 慢性粒-单核细胞白血病（CMML）特异性预后积分系统（CPSS）

预后参数	积分		
	0	1	2
WHO分型	CMML-1	CMML-2	–
FAB分型	MD-CMML	MP-CMML	–
CMML特定的细胞遗传学危度分层^a^	低危	中危	高危
红细胞输注依赖	否	是	–

**注** MD-CMML：骨髓增生异常型CMML；MP-CMML：骨髓增殖型CMML。^a^+8、−7/7q−或复杂核型为高危组，正常核型或−Y为低危组，除外高危和低危的所有染色体异常归为中危组。根据总积分将患者分为4组：低危（0分）、中危1（1分）、中危2（2～3分）和高危（4～5分）

**表4:** 慢性粒-单核细胞白血病（CMML）遗传学预后参数积分

遗传学预后参数	积分		
	0	1	2
CPSS细胞遗传学预后分组^a^	低危	中危	高危
ASXL1	野生型	突变型	
NRAS	野生型	突变型	
RUNX1	野生型		突变型
SETBP1	野生型	突变型	

**注** ^a^+8、−7/7q−或复杂核型为高危组，正常核型或−Y为低危组，除外高危和低危的所有染色体异常归为中危组

最近Teffri等[15]–[16]提出了BLAST和BLAST-Mol积分系统。BLAST积分系统包括3个参数：外周血细胞分类计数原始细胞≥2％（1分）、WBC≥13×10^9^/L（1分）、中度贫血［HGB 90～<110 g/L（男性）和80～<100 g/L（女性）］（1分）或重度贫血［输血依赖或HGB<90 g/L（男性）和<80 g/L（女性）］（2分），将患者分为低危（0分）、中危（1分）和高危（2～4分），中位OS时间分别为63、28和13个月。BLAST-Mol积分系统增加了染色体核型和基因突变。预后不良染色体核型是指除−Y和+8以外的所有染色体核型异常。预后良好突变基因包括TET2和PHF6，不良预后突变基因包括DNMT3A、U2AF1、BCOR、SETBP1、ASXL1、NRAS、PTPN11、RUNX1和TP53。结合染色体核型和基因突变进行分子学预后分组：低危（有预后良好异常且没有预后不良异常）、中危（没有预后良好异常）和高危（有预后不良异常且没有预后良好异常）。最后将高危分子学（2分）、中危分子学（1分）、外周血细胞分类计数原始细胞≥2％（1分）、WBC≥13×10^9^/L（1分）、中度贫血（1分）或重度贫血（2分）整合将患者分为低危（0～1分）、中危（2～3分）和高危（4～6分）3组，OS时间分别为74、23和12个月。BLAST-Mol的预后效力好于CPSS-Mol，但其在患者临床治疗决策中的应用尚无相关研究[15]。

对于确诊的CMML患者，在制定治疗方案前应按CPSS或BLAST进行预后危度分组，如果患者同时有细胞遗传学和基因突变检测资料，则应按CPSS-Mol或BLAST-Mol进行预后危度分组。例2 CPSS为中危1，CPSS-Mol为高危，BLAST为中危，BLAST-Mol为高危。

在临床实践中，面对CMML患者需要我们处理的主要问题有：①有症状的血细胞减少：如需要输血的贫血，或出现影响生活质量的症状性贫血（如疲劳、呼吸困难）；血小板计数过低导致出血，或血小板计数呈进行性、显著下降（通常<50×10⁹/L，或伴有活动性出血）；重度中性粒细胞减少导致反复发生或严重的感染。②骨髓高增殖导致的症状负荷：单核细胞/白细胞增多导致器官浸润并引发症状（如脾肿大引起早饱感、腹痛或门静脉高压；皮肤浸润；齿龈增生等）；WBC增高［（>25～50）×10⁹/L］，尤其当伴有原始细胞增多时，可能存在白细胞淤滞的风险。③疾病进展/转化。④因脾肿大出现如左上腹疼痛、饱胀感、进食减少等，肝脏肿大引起的不适，或罕见的皮肤、中枢神经系统浸润症状。⑤显著的全身性症状或炎症表现：无法解释的、与疾病相关的严重症状（如持续性或反复发热、显著的盗汗、非预期的体重减轻、严重的疲劳或体能状态下降）；自身免疫或炎症表现：如浆膜炎（胸膜炎、心包炎）、关节炎或血管炎，且对常规对症治疗反应不佳。因此，在给CMML患者制定治疗方案时应综合评估症状、疾病负荷（血细胞减少或增殖表现）、疾病进展证据以及患者的个体状况和意愿，而不仅仅是依赖预后评分。治疗的主要目标是控制症状、改善血细胞计数、减轻疾病负荷（如缩小肿大器官）、延缓疾病进展，并最终提高患者的生活质量。

最佳支持治疗是所有患者的基础治疗。症状性贫血患者，依血清EPO水平可选择EPO或罗特西普，有出血症状的血小板减低患者可选择TPO受体激动剂如艾曲泊帕，发热性中性粒细胞缺乏的患者可短期使用G-CSF。高白细胞伴粘滞症状患者可选择白细胞单采联合口服羟基脲降白细胞，必要时可采用低剂量阿糖胞苷治疗。炎性免疫症状明显的患者可加用糖皮质激素治疗。

异基因造血干细胞移植（allo-HSCT）是迄今唯一可能治愈CMML的方法。CPSS-Mol高危患者，应首选allo-HSCT。CPSS-Mol中危2患者，如果骨髓和（或）外周血原始细胞计数高、红细胞和（或）血小板输注依赖、存在CPSS-Mol未包含的高危基因突变或基因突变数量多，以及既往治疗后复发或难治，也应尽早考虑allo-HSCT。CPSS-Mol中危1和低危患者一旦疾病评估发现疾病进展亦应考虑allo-HSCT[1]–[3]。关于移植前使用诱导化疗或去甲基化药物进行减瘤治疗是否能改善预后，目前存在争议。如果能够及时找到合适供者并进行移植，则不推荐进行移植前治疗，以避免筛选出耐药的CMML克隆。现有研究[1]–[3]提示供者类型（全相合同胞、全相合无关供者、单倍体相合供者或无关脐带血）以及干细胞来源对移植疗效均无显著影响。预处理强度应基于个体情况，综合考虑疾病生物学特征、患者年龄和体能状况等来加以选择。

去甲基化药物是非HSCT治疗的骨架药物，骨髓增生异常型可选用阿扎胞苷（75 mg·m^−2^·d^−1^，连用7 d）或地西他滨（20 mg·m^−2^·d^−1^，连用5 d），地西他滨对骨髓增殖型疗效好于阿扎胞苷，原始细胞增高患者可选用阿扎胞苷/地西他滨联合维奈克拉（400 mg/d，连用14 d）。

例2为年轻患者，CPSS-Mol和BLAST-Mol均为高危组，患者与其父亲HLA配型8/12相合，2022年6月13日于我院移植中心行单倍体造血干细胞移植。2025年11月3日门诊复查，血常规：WBC 4.17×10^9^/L，RBC 5.46×10^12^/L，HGB 173 g/L，PLT 104×10^9^/L，单核细胞12.2％，粒细胞72.3％，AMoC 0.51×10^9^/L，ANC 3.01×10^9^/L。骨髓细胞形态学无明显异常，流式细胞术可检测残留病阴性。患者至今仍处于完全缓解，恢复了正常工作和生活。
